# Supplementary material for: EnzML: multi-label prediction of enzyme classes using InterPro signatures
Source: BMC Bioinformatics. 2012 Apr 25;13:61. doi: 10.1186/1471-2105-13-61 (PMC3483700; doi:10.1186/1471-2105-13-61)
Supplement: Addtional file 5 — The Java code to format the data files, evaluate and predict. The file enzml_java_code.tar.gz contains the Java code used to format database data to ARFF and XML formats, to execute cross and train-test (jackknife) evaluations and to record evaluation results to database. More information is included in the readme.txt file and the Javadoc files. The code can be used with a MySQL database. To use a different database software, other JDBC drivers might be required. [file 1471-2105-13-61-S5.gz › java_code/enzml2011/doc/uk/ac/ed/inf/csb/taweka/mulan/package-use.html]

Uses of Package uk.ac.ed.inf.csb.taweka.mulan


---


|  |  |  |
| --- | --- | --- |
| **Overview** \| **Package** \| Class \| **Use** \| **Tree** \| **Deprecated** \| **Index** \| **Help** | |  |
| PREV   NEXT | **FRAMES**    **NO FRAMES**     **All Classes** |  |


---


## **Uses of Package uk.ac.ed.inf.csb.taweka.mulan**

| Packages that use uk.ac.ed.inf.csb.taweka.mulan | |
| --- | --- |
| **uk.ac.ed.inf.csb.taweka.mulan** |  |
| **uk.ac.ed.inf.csb.taweka.test.mulan** |  |

Classes in uk.ac.ed.inf.csb.taweka.mulan used by uk.ac.ed.inf.csb.taweka.mulan

****MulanArffGenerator****
  
          Class

****MulanDataSetWriter****
  
          This class generates a data set (instances, attributes, classes) in Mulan
style (for multi-label machine learning).

Classes in uk.ac.ed.inf.csb.taweka.mulan used by uk.ac.ed.inf.csb.taweka.test.mulan

****MulanArffGenerator****
  
          Class

****MulanDataSetWriter****
  
          This class generates a data set (instances, attributes, classes) in Mulan
style (for multi-label machine learning).

****MulanDbDataLoader****
  
          Loads data from database to a Mulan data factory

****MulanInstancesFactory****
  
          Class

---


|  |  |  |
| --- | --- | --- |
| **Overview** \| **Package** \| Class \| **Use** \| **Tree** \| **Deprecated** \| **Index** \| **Help** | |  |
| PREV   NEXT | **FRAMES**    **NO FRAMES**     **All Classes** |  |


---
